# Facial Expression Processing Is Not Affected by Parkinson’s Disease, but by Age-Related Factors

**DOI:** 10.3389/fpsyg.2019.02458

**Published:** 2019-11-14

**Authors:** Dilara Derya, June Kang, Do-Young Kwon, Christian Wallraven

**Affiliations:** ^1^Department of Brain and Cognitive Engineering, Korea University, Seoul, South Korea; ^2^Department of Neurology, Korea University Ansan Hospital, Korea University College of Medicine, Ansan-si, South Korea; ^3^Department of Artificial Intelligence, Korea University, Seoul, South Korea

**Keywords:** facial expressions, Parkinson’s disease, aging, conversational expressions, positivity, rating

## Abstract

The question whether facial expression processing may be impaired in Parkinson’s disease (PD) patients so far has yielded equivocal results – existing studies, however, have focused on testing expression processing in recognition tasks with static images of six standard, emotional facial expressions. Given that non-verbal communication contains both emotional and non-emotional, conversational expressions and that input to the brain is usually dynamic, here we address the question of potential facial expression processing differences in a novel format: we test a range of conversational and emotional, dynamic facial expressions in three groups – PD patients (*n* = 20), age- and education-matched older healthy controls (*n* = 20), and younger adult healthy controls (*n* = 20). This setup allows us to address both effects of PD and age-related differences. We employed a rating task for all groups in which 12 rating dimensions were used to assess evaluative processing of 27 expression videos from six different actors. We found that ratings overall were consistent across groups with several rating dimensions (such as arousal or outgoingness) having a strong correlation with the expressions’ motion energy content as measured by optic flow analysis. Most importantly, we found that the PD group did not differ in any rating dimension from the older healthy control group (HCG), indicating highly similar evaluation processing. Both older groups, however, did show significant differences for several rating scales in comparison with the younger adults HCG. Looking more closely, older participants rated negative expressions compared to the younger participants as more positive, but also as less natural, persuasive, empathic, and sincere. We interpret these findings in the context of the positivity effect and in-group processing advantages. Overall, our findings do not support strong processing deficits due to PD, but rather point to age-related differences in facial expression processing.

## Introduction

Human communication is rich with both verbal and non-verbal signals, enabling us to act and interact in highly complex social situations. Among the non-verbal signals, facial expressions are one of the most important channels of communication. Facial expressions are produced by facial muscle movements and communicate across a whole range of signal types – from strong and deep-rooted survival states up to very subtle communicative signals, such as an eye-brow raise that are typically used in a conversational context (e.g., Kaulard et al., 2012). Expressions have been studied since at least Charles Darwin’s time (Darwin and Prodger, 1998) and have become a core topic in psychological research after the studies of Ekman and Friesen (1971) during the 1960s. In these studies, several emotional expressions were tested across different literate and pre-literature cultures and it was found that six facial expressions were recognized equally well in all cultures – these are anger, disgust, fear, happy, sad, and surprised and have since also become known as the “basic” or “universal” facial expressions (Ekman and Friesen, 1971) as well as coded in terms of specific muscle movements the Facial Action Coding System (FACS) (Ekman and Friesen, 1978). Although these six expressions are referred to as “universal,” various studies have reported differences in perception and production of facial expressions depending on gender (Ebner et al., 2010), cultural and ethnic background (Matsumoto, 1993; Jack et al., 2009, 2012a,b; Jack, 2013), as well as age (Ebner et al., 2010). Concerning the latter, age-related factors, for example, several studies have found a “positivity effect” in which negative and neutral facial expressions are evaluated as being more positive by older viewers compared to younger ones (Calder et al., 2003; Isaacowitz et al., 2007; Ebner, 2008; Ebner et al., 2011, 2013) but the age-related factor is not only applicable for older seeing younger expressions, also applies for own age negative expressions being less memorable for both young and older adults (Ebner and Johnson, 2009). Facial expressions over a lifespan alter in both recognition and overall processing, it has been reported performance of recognition improves from childhood to early adulthood and starts to decline in later adulthood (Mill et al., 2009; Williams et al., 2009). Performance difference in older and younger adults have been suggested to be caused by attention preference of older people. This attention difference is due to motivational orientation, advantaging positive input in consideration of time perspective (Carstensen, 1992; Hugenberg et al., 2013). Another age-related aspect affecting facial expression processing has been argued to be alterations of frontal brain areas and the amygdala influencing the processing of anger, sadness, and fear (Borod et al., 2004). Hence, there are significant age-related differences that modulate the perception and production of facial expressions.

Processing of facial expressions is not only affected by these above-mentioned factors, but also may be influenced by neurological and neurodegenerative diseases. In the present study, we focus on Parkinson’s disease (PD), which is a neurodegenerative movement disorder caused by the loss of neuronal cells in the basal ganglia resulting among other effects in a large decrease in dopamine-secreting neurons. The disease affects motor and cognitive functions of the patient. Another key symptom of PD is hypophonia, which reduces patients’ vocal intensity and decreases voice quality by giving the patient a quieter, less clear voice (Hartelius and Svensson, 1994). PD also adversely affects patients’ ability to find the right words and results in halting speech. In the context of facial expression production, another secondary symptom of Parkinson’s is hypomimia, a muscle rigidity that results in a “masked,” expressionless face, restricting patients’ ability to use non-verbal expression communication (Gibb and Lees, 1988; Hughes et al., 1992; Aarsland et al., 1999; Jankovic, 2008).

Impaired *production* of facial expressions leads to the question whether there may be impaired *perception* as well. The “facial feedback hypothesis” provides a framework for such an effect, positing that facial activity influences emotional experience (Strack et al., 1988; Davis et al., 2010; Gray and Tickle-Degnen, 2010; Ricciardi et al., 2017). More specifically, knowledge about facial expression is stored as a sensorimotor simulation that is activated during both production and perception. Hence, emotion perception can be understood as multi-modal integration in which the sensorimotor knowledge influences visual perception and generates predictions (Wood et al., 2016). Since PD patients have impaired sensorimotor loops, the facial feedback hypothesis would posit that this in turn will also influence perception: indeed, some studies have reported that people with PD have deficits in recognizing certain facial expressions such as fear, anger, and disgust (Adolphs et al., 1994; Calder, 1996; Kan et al., 2002; Sprengelmeyer et al., 2003; Lawrence et al., 2007; Marneweck et al., 2014). However, other studies (Madeley et al., 1995; Adolphs et al., 1998; Wieser et al., 2012) have found no such impairments in emotional facial expression recognition (see section “Discussion” for a more thorough discussion of the studies related to this topic).

In addition to the open question whether or not PD patients have impaired face expression processing, these previous studies have two important limitations: first, they solely used static stimulus presentation and, second, they relied only on the six basic facial expressions. Concerning the first point, a series of recent studies has shown, however, that perception of facial expressions when presented in their real-life dynamic form (Bülthoff et al., 2011), results in significantly different performance patterns (Ambadar et al., 2005; Cunningham and Wallraven, 2009) as well as involvement of different brain areas (Kilts et al., 2003; Sato et al., 2004; Yoshikawa and Sato, 2006; Trautmann et al., 2009; Perdikis et al., 2017) compared to processing of static expressions. The second, important aspect that has been neglected in previous studies is that in daily life, expressions do not only consist of the six basic expressions, but they also include a much wider range of communicational, conversational, and emotional expressions and facial gestures such as tiredness, boredom, flirting, etc. (Cunningham et al., 2005; Nusseck et al., 2008; Kaulard et al., 2012; Lee et al., 2012). With respect to PD, therefore little is actually known about how the presence of this neurodegenerative condition may affect perception and processing of the wider and perhaps ecologically more valid (Russell, 1994) range of facial communication.

In summary, the aim of the present study is to overcome limitations in the existing literature on facial expression processing by investigating the effects of age and presence of PD on a wider range of dynamic, emotional and conversational facial expressions. Specifically, we will test and compare three participant populations: to investigate the variable of age, we compare facial expression processing in a younger healthy group with that in an older healthy group; to investigate the variable of PD, we compare expression processing in a PD group with the (age-matched) older health group. Under the assumption of the facial feedback hypothesis, we would predict that due to the presence of muscle movement impairments, processing differences would occur in the PD group versus the older participant group. Additionally, the positivity effect would see additional differences due to the variable of age that would be seen most clearly when contrasting the older healthy group versus the younger healthy group. This study sets out to compare and contrast these two potential effects.

## Materials and Methods

### Participants

All individuals provided written informed consent to participate in this study and the nature of the study was explained to each participant. All participants were paid for their participation and were informed about the possibility to stop the experiment anytime they would like to. The experiment was conducted in accordance with the Declaration of Helsinki 1963 and approved by the Ethics Committee of Korea University (AS17006).

To investigate the influence of PD on facial expression processing, we recruited a first group of 20 individuals (10 male, 10 female) with non-demented PD (group PD). Twenty healthy controls (group HC, 7 male, 13 female) matched for age, sex, and intellectual level were recruited for a matched control group [group HC; mean age 59.6 ± 7.1 (range: 46–73 years)]. To investigate the variable of age, we recruited a second control group (group HCS) consisting of 20 participants (10 male, 10 female) of university students ranging between first year and fourth year of university [mean 23.3 ± 2.8 (range: 19–30 years)]. Participants in both control groups had no history of stroke and no symptoms of neurological or other psychiatric disorders. All participants had normal or corrected-to-normal vision. The patient population was recruited from the Neurology Department at the Korea University Ansan Hospital where they had been diagnosed by a resident neurologist.

The mean age of the patient group was 58.5 ± 8.4 years (range: 47–74 years) with a disease duration range of 9 – 4 years. The severity of Parkinson symptoms was equivalent to level II or III on the Hoehn and Yahr (1967) scale. In order to rule out medication influence, all PD participants were asked to abstain from taking their medication the night before the experiment (Sprengelmeyer et al., 2003; Lawrence et al., 2007).

The two older participant groups were matched in terms of their age distribution (Mann–Whitney *U* Test *U* = 183.000, *p* = 0.654, *r* = 0.073). All groups were matched as best as possible in terms of their education level although the HCS group had a somewhat higher average education level compared to the two older groups (both *p* < 0.05) – see Table 1 for further data on both populations.

**TABLE 1:** Clinical characteristics.

**Variable**	**PD (*n* = 20)**	**HC (non-PD) (*n* = 20)**	**HCS (*n* = 20)**
Sex (F/M)	10/10	13/7	10/10
Age (Years)	58.5 ± 8.4	59.6 ± 7.1	23.3 ± 2.8
Education (Years)	11.3 ± 4.6	12.5 ± 3.1	14–18 years
UPDRSIII	23.9 ± 9.3	N/A	N/A
Disease duration (Years)	5.5	N/A	N/A
DA intake (mg/day)	3.2	N/A	N/A
LEDD L-dopa intake (mg/day)	405.1	N/A	N/A
BDI	12.5 ± 8.6	N/A	N/A
MMSE	27.4 ± 1.7	N/A	N/A
MoCA-K	25.0 ± 3.6	N/A	N/A
STAI	43.8	N/A	N/A
Hoehn and Yahr rating scale	1.8 ± 0.5	N/A	N/A

*Values are mean ± SD. PD, Parkinson’s disease; HC (non-PD), healthy control; F, female, M, male; UPDRSIII, Unified Parkinson’s Disease Rating Scale Part III motor score; DA, dopamine agonist; LEDD, levodopa equivalent; BDI, Beck Depression Inventory; MMSE, Mini-Mental State Examination (MMSE max. = 30, MMSE min. = 25; MoCA-K, Korean Version of the Montreal Cognitive Assessments (MoCA-K max. = 30, MoCA-K min. = 16); STAI, State trait anxiety inventory.*

### Stimuli

To conduct research on conversational expressions, an appropriate database is required. Since existing databases usually focus only on a few, emotional facial expressions and mostly contain only static stimuli, we used a newly developed, validated facial expression database [the KU Facial Expression Database: Lee et al. (2012) based on protocols established with the MPI Facial Expression Database (Kaulard et al., 2012)]. The database contains more than 50 facial expressions performed by 20 native participants (referred to as “actors” in the following, although none of the participants had acting experience). The following briefly describes the recording methods that were employed.

In order to ensure a good compromise between fully scripted (but potentially posed and unnatural) and unscripted (natural, but non-controlled) expressions, a method-acting protocol was used during the recordings. For this, the experimenter read a developed scenario containing a short description of an event to the actor and asked them to imagine themselves in the scenario and to react accordingly. This process was repeated three times to yield three repetitions of each expression. The scenarios were designed to accommodate a large range of different emotional and conversational contexts. Importantly, they were also created with a conceptual hierarchy in mind: for example, there are many types of smile (pure smile, sad smile, reluctant smile, flirtatious smile…) or many types of agreement (pure agreement, considered agreement, reluctant agreement…). The full list of expressions and scenarios can be found in Kaulard et al. (2012). To keep the following experiments with elderly participants at a reasonable duration, we selected 27 facial expressions spanning a wide variety of communicative and emotional signals of the three most consistently rated actors for each expression as our stimuli. These 27 expressions are listed in the Table 2.

**TABLE 2:** Facial expression scenarios used in the present study.

**Explanation of scenario**	**Abbreviations**
Showing a considered agreement	agcons
“Aha” moment (when one suddenly understands something)	aha
Anger	ang
Arrogant (looking down on somebody)	arr
Being bothered by something	bot
Showing contempt toward someone	cont
Showing that one is not interested (“I don’t care!”)	dcar
Disagreeing with something	disag
Being disgusted	disg
Being embarrassed	emb
Reacting in an evasive manner	eva
Feeling fearful (terrified of something)	feter
A genuine happy laugh	halau
A satiated smile (as if after a good meal)	hasa
Imagining something negative	imneg
Being impressed by something	impr
Feeling insecure	ins
Feeling compassion toward someone	mitl
Feeling pain	paf
Being irritated (“rolling your eyes”)	paf
Remembering something neutral	reneu
Being sad	sad
A flirtatious smile	smfli
A reluctant smile	smrel
A sardonic smile	smsa
A sad/nostalgic smile	smsad
Being tired	tir

The overall procedure for stimulus validation followed that of Kaulard et al. (2012) and Lee et al. (2012) and was based on an experiment in which video clips of 10 actors and 57 expressions were shown in random order to 14 participants, who were asked to freely describe the expression that they contained. The data from this experiment was then reviewed by three independent raters, who were given the desired expression label (shown in Table 1) and were asked to judge for each answer whether it conformed to the label or not. Here, we used a subset of this data, focusing on 27 expressions spanning a wide range of conversational and emotion signals. For each expression, we next selected three actors for whom the validation rate of the three raters was among the highest – overall, this validation rate was 77.8% for the expressions tested here. Note, that the task for participants in this experiment was not a forced-choice task as typically reported, but rather a free association task which explains the lower percentage ratings (for example, Russell, 1994).

### Procedure and Task

The task consisted of a standard rating task in which each participant was asked to perform evaluative ratings of the 27 different facial expressions from the three different actors. In each of the 81 resulting trials, a video sequence was shown to participants, after which they were to rate each expression according to 12 different dimensions: arousal = the intensity of the expression, attractiveness = how attractive the facial expressions (not the person) was displayed, dynamics = the amount of motion contained in the expression, empathy = whether the expression makes the observer feel empathic, familiarity = whether this is a typical expression, intelligence = the degree of intelligence of the facial expression, naturalness = whether this was a posed or a natural expression, outgoingness = the degree of extroversion, persuasiveness = the degree to which the expression can persuade the observer, politeness = the degree of politeness of the facial expression, sincerity = whether the expression was meant in a sincere fashion, valence = whether the expression was positive or negative.

These rating dimensions were selected based on prior experiments about ratings of emotional and evaluative concepts and were designed to cover a wide range of communication aspects (Fontaine et al., 2007; Castillo et al., 2014).

Ratings were done on a 7-point Likert-type scale (1 = does not convey the property at all – 7 = fully conveys the property, except for valence where 1 = fully negative – 7 = fully positive). Each PD individual completed the task in three sessions due to being withdrawn from medication the night before the experiment, the HC and HCS groups completed the task in one session.

### Experimental Setup

The dynamic stimuli of the facial expressions were shown to the participants on a 15-inch high-resolution monitor placed at a distance of 60 cm in a quiet laboratory. Each participant was informed that they were allowed to re-play the video sequence as many times as they would like to (this option was not exercised by the HCS group, whereas the HC and PD group used the option only during the first maximum of five trials to get adjusted to the experimental procedure). The order of the stimuli was randomized differently for each participant. All participant groups (PD, HC, and HCS) rated each video sequence on a paper-based questionnaire containing the 12 dimensions. There was no time limit set for the task. All groups were given opportunities for breaks to avoid fatigue – the HCS groups finished the ratings in around 60 min, whereas both older groups took a total of around 90 min.

### Statistical Analyses

Associations between dimensions were determined using mixed ANOVAs, Pearson correlations, two-tailed *t*-tests, or non-parametric Wilcoxon tests where applicable. All three groups were analyzed for within-group reliability and across-group reliability by means of correlations and compared with bootstrapped confidence intervals. In addition, a factor analysis was conducted for all the groups for inter-dependencies among correlated dimensions. To further analyze the overall findings and set them into context, optical flow estimation was conducted on all video sequences and correlated with the behavioral ratings. All data analyses were performed using standard statistical functions in MATLAB (R2014a, The MathWorks, Natick, MA, United States).

## Results

### Variability of Ratings

The first analysis was concerned with assessing the *absolute* variability of the rating values across the three tested groups, that is, how the raw ratings gathered from participants varied for each of the ratings dimensions and in each of the groups.

**FIGURE 1:**
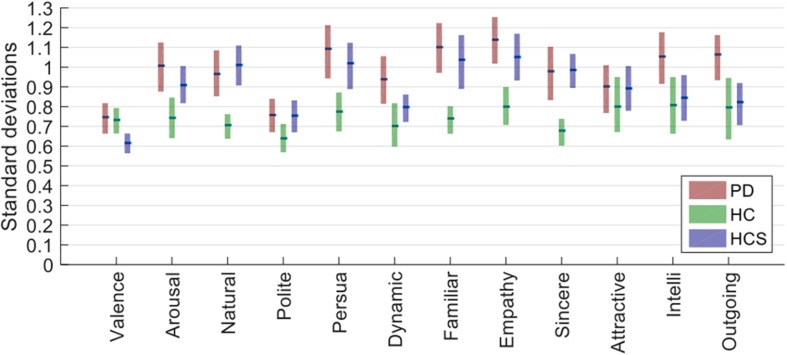
Standard deviations for the three groups (averaged across actors first and then determined for participants and averaged across all 27 expressions). Bars show the estimated median of the bootstrapped sample distribution, and the boxes show the 95% confidence intervals estimated from bootstrapping.

For this, we first averaged data across actors for each participant, then took the resulting data matrix (containing number of participants × number of expressions × number of rating dimensions cells) and determined the standard deviation across participants for each combination of expression and rating. Next, we averaged the standard deviations for all 27 expressions and compared the average variability for each rating dimension across the three groups (see Figure 1). We performed a two-way mixed ANOVA with within-participant factor *rating dimensions* and between-participant factor *participant group*. Both main effects were significant [*F*(2,627) = 12.721, *p* < 0.001, *n*^2^ = 0.308; *F*(11,627) = 21.736, *p* < 0.001, *n*^2^ = 0.261] as well as their interaction [*F*(22,627) = 1.943, *p* = 0.006, *n*^2^ = 0.048]. Following up on the latter interaction, the HC group had the lowest standard deviation (median SD_HC = 0.740) compared to the two other groups as compared by Mann–Whitney-*U* tests (mean SD_PD = 0.991, *U* = 11.000, *Z* = 3.522, *p* < 0.000, *r* = 0.719; mean SD_HCS = 0.905, *U* = 19.000, *Z* = 3.060, *p* = 0.002, *r* = 0.625).

Given that ratings were done on a 7-point scale, a standard deviation of roughly 1 rating point on average is within reasonable bounds – especially considering that there were no explicit anchoring instructions given to participants (see next analysis). Although the PD group had the highest standard deviation, this was actually comparable to that of the younger healthy control group (HCS), showing that neither age nor disease prevalence provides a simple explanation for the results.

### Reliability of Ratings Within Groups

The previous analysis focused on comparing the ratings in absolute terms. However, in rating tasks without an explicit anchoring phase it is possible that participants may have chosen different anchoring points for their scales (e.g., Fontaine et al., 2007; Castillo et al., 2014), that is, what “fully positive” means for one person may be different from another person. To address this issue, the next analysis focused on comparing the relative consistency within each participant group by means of correlations.

To assess this *within-dimension reliability* we performed Pearson correlations, correlating the rating responses across participants, but separately for each rated dimension. Confidence intervals (95%) were obtained by a standard bootstrapping procedure with 1000 samples. Fisher’s z’ transformation was applied to convert r’s to normally distributed z’.

**FIGURE 2:**
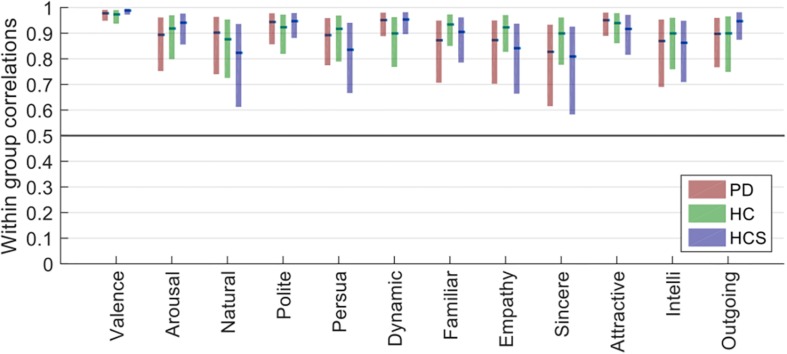
Plots of the within-group reliability for each dimension of the three participant groups (bars show the estimated median of the bootstrapped sample distribution, and the boxes show the 95% confidence intervals estimated from bootstrapping).

As Figure 2 shows, all confidence intervals are well above *r* = 0.5, indicating high inter-rater reliability. Furthermore, confidence intervals for all groups overlap for each of the tested dimensions, showing similar rating reliabilities across the three participant groups (all Mann–Whitney-*U* tests *p* > 0.2). Compared to the previous analysis of *absolute* rating variability, here all groups showed similar *relative* rating behavior. This finding demonstrates that different groups for some dimensions may have used different absolute anchoring points for their ratings, but also that overall participants agreed well on increases or decreases in the evaluative ratings *relative* to these anchoring points.

Additionally, of all the tested dimensions, valence had the lowest variability in reliability, as well as the highest overall reliability (median *r* > 0.97 for all groups).

### Factor Analysis for Each Participant Group

Since the ratings were done along 12 dimensions, we next tried to identify sets of combined factors among the potentially correlated rating dimensions. To this end, we implemented an exploratory factor analysis that exploits correlational structure in the data to determine such sets, resulting in a smaller number of factors each of which consists of several rating dimensions “loading onto” that factor with different correlation strength. If the rating pattern in one group would be very different from that of another group, the factor analysis for that group should recover factors containing different combinations of the rating dimensions compared to those of another group.

**FIGURE 3:**
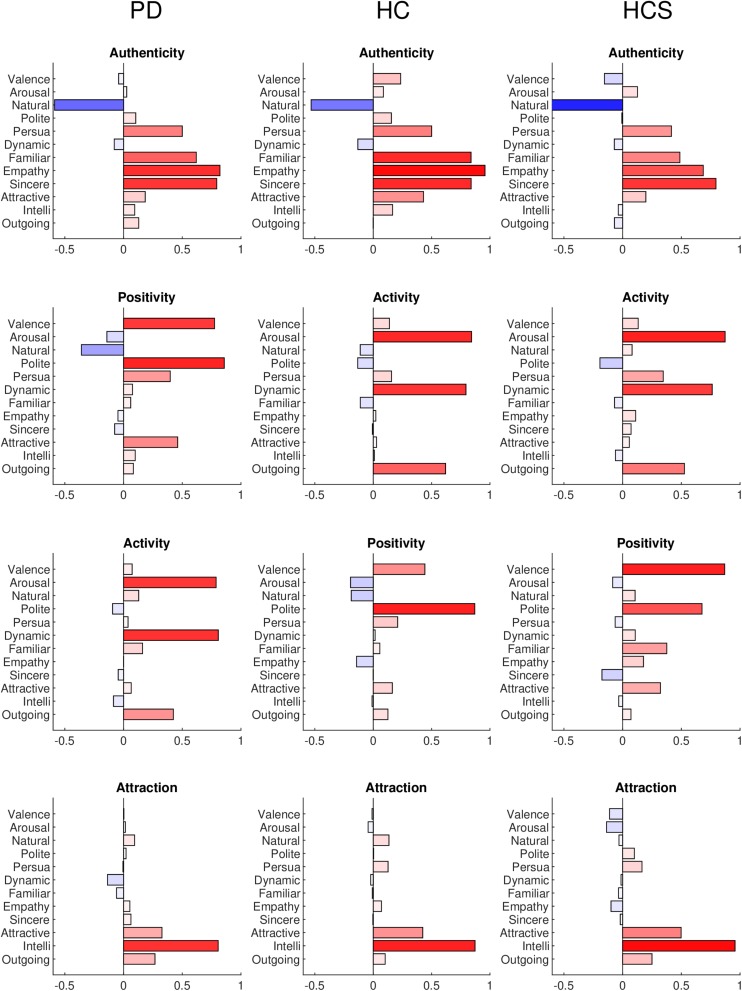
Factor analysis results for the PD, HC, and HCS groups for all 12 dimensions. Each loading is plotted as positive (red) or negative (blue) with color saturation indicating the strength of the loading.

In the following analyses, we used the full data matrix (number of participants × number of expressions × number of rating dimensions) as input to the “factoran” method in Matlab. A “promax” variance-based rotation criterion was applied to maximize the score loadings in the factor analysis. The optimal number of factors was determined by parallel analysis (PA) (Ledesma and Valero-Mora, 2007), which determines the number of eigenvalues that are larger than those obtained by randomly permuting the data. This value was four for the HCS group and between three and four for both older groups, such that we set the total number to four for easier comparison between all groups. The results of the factor analyses are shown in Figure 3 for the three groups with names given to each of the four factors based on the significantly loading dimensions. The factors are sorted in terms of their overall importance from high to low.

The factor analysis of the PD group results indicates familiarity (*r* = 0.6209), empathy (*r* = 0.8282), and sincerity (*r* = 0.7961) in contrast to naturalness (*r* = −0.5865) as a first factor. We termed this factor “authenticity” (note, that naturalness was phrased in an opposite way on the Likert-scale). The second factor has high loadings of valence (*r* = 0.7851), and politeness (*r* = 0.8607), which seems related to positivity and assertiveness, so that we termed this factor as “positivity.” The third factor combines arousal (*r* = 0.7939), dynamics (*r* = 0.8016), and to a lesser degree outgoingness (*r* = 0.4294), all of which relate to “activity” in the facial expression. Finally, the fourth factor combines intelligence (*r* = 0.8294) with weaker contributions of attractiveness (*r* = 0.3114), and outgoingness (*r* = 0.2571), a cluster that we termed “attraction” to the facial expression.

The factor analysis of the HC group is similar to the PD group results. Again, the first factor contrasts familiarity (*r* = 0.8382), empathy (*r* = 0.9576), and sincerity (*r* = 0.8393) with naturalness (*r* = −0.5320), again constituting the authenticity factor. Compared to the PD group, the second (“activity,” arousal *r* = 0.8430; dynamics *r* = 0.7947; outgoingness *r* = 0.6203) and third factor (“positivity,” valence *r* = 0.4427; politeness *r* = 0. 8703) switch places, but retain their dimension loadings. The fourth factor again consists of attraction with similar contributions (intelligence *r* = 0.8725; attractiveness *r* = 0.4243; outgoingness *r* = 0.1023).

Finally, the factor analysis of the HCS group shows similar factors to both older groups: authenticity (naturalness *r* = −0.8641; familiarity *r* = 0.4875; empathy *r* = 0.6869; sincerity *r* = 0.7949), activity (arousal *r* = 0.8730; dynamics *r* = 0.7630; outgoingness *r* = 0.5260), positivity (valence *r* = 0.8696; politeness *r* = 0. 6763), and attraction (intelligence *r* = 0.9576; attractiveness *r* = 0.4982; outgoingness *r* = 0.2502) factors were recovered from the ratings.

Overall, the factor analyses confirm the previous results inasmuch as they are able to identify broadly similar evaluation patterns for our diverse range of facial expressions across all three groups using four evaluation factors. In addition, the relative loadings of the rating dimensions onto the factors are similar for each of the participant groups.

### Correlations of Ratings Across Groups

The previous factor analyses uncovered similar factors across groups, implicating that the broad correlational structure within each group is similar. To assess this correlational structure in more detail across groups, the next analysis conducted correlations separately for each of the rating dimensions in a way similar to the within-group analysis, except that correlations were now determined in a between-group analysis across all *pairs of groups* for 1000 random permutations in a bootstrap analysis.

**FIGURE 4:**
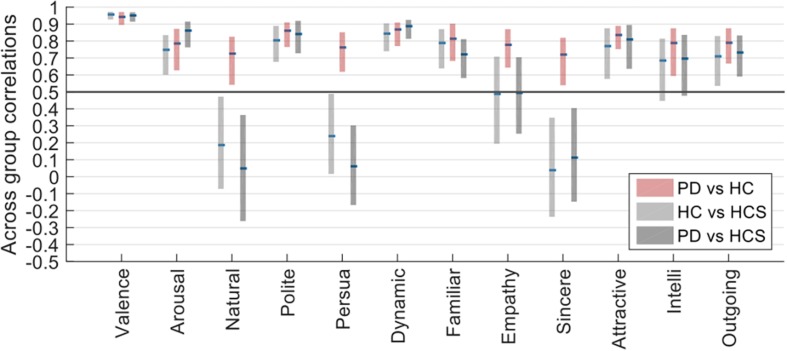
Across-group correlations of HC vs. PD, HCS vs. PD, and HC vs. HCS.

Figure 4 shows that despite similar within-group reliabilities (Figure 2), several across-group comparisons have significantly lower correlations. In particular, whereas all comparisons *within* the same age-group (namely for PD versus HC, again see Figure 2) are above *r* = 0.5, several comparisons *across* age groups (namely for HC versus HCS, and PD versus HCS, Figure 4) are significantly lower. This relates to the dimensions of naturalness, persuasiveness, sincerity, and – to a somewhat lesser extent – empathy. For these four dimensions, ratings are highly consistent within the same age group but much less consistent across age compared to the other rating dimensions which show similar consistency. Hence, evaluation of facial expressions at least for these dimensions is not affected by prevalence of Parkinson’s, but rather by the variable of age.

These results were confirmed by a mixed ANOVA on the Fisher-z-transformed correlation values, for which the interaction of within-participant (rating dimension) and between-participant factor (across-group comparison) became highly significant [*F*(22,32967) = 1781.268, *p* < 0.001, n2 = 0.178].

**FIGURE 5:**
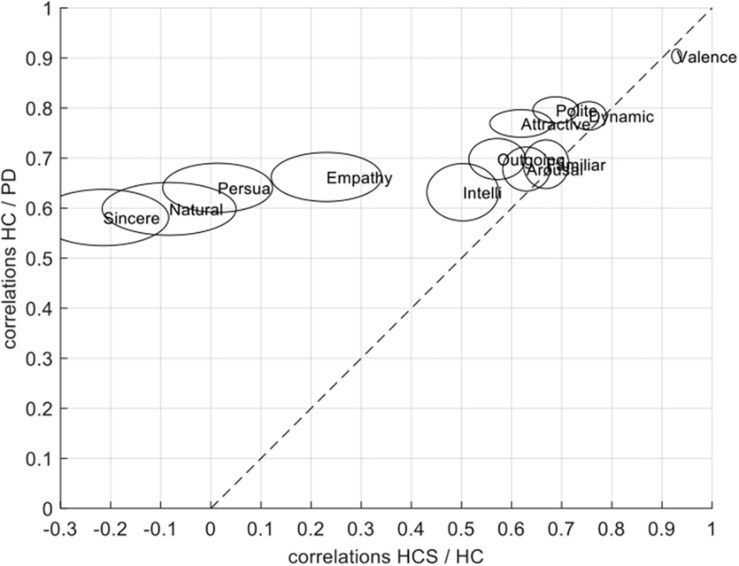
Scatter plot showing the relationship between across-group correlations for HC/PD correlations and HCS/PD correlations for the jointly rated evaluation dimensions. The size of each circle is proportional to the average variability of the correlations as determined in the split-half procedure.

The difference in correlation structure of across group correlations of HC/PD and HC/HCS can also be seen in the scatter plot in Figure 5. In this plot, the correlations between HC and PD are on the *y*-axis, and the correlations between HCS and HC are on the *x*-axis. If all groups would have similar relationships across ratings, we would expect all data points to lie on the diagonal. Similarly, if data points are above the diagonal, the two older participant groups (factor *disease*) are more similar than the younger to the older group (factor *age*). Conversely if most data points are below the diagonal, the effect of disease is stronger. Since most ratings lie above the diagonal, however, this means that on average, correlations between the two older participant groups are more similar than those between the patient group and the younger group.

### Optical Flow and Motion Analysis

In order to investigate to what degree the ratings may be linked to and hence potentially explained by (lower-level) movement in the picture, we next performed an optical flow analysis. Such an analysis computes the pattern of the movement of a presented video by calculating the magnitude and the direction of motion changes during a time interval and estimates the pixel flow (position) in the next frame.

We used the optical flow method to estimate the motion energy of each of the 27 facial expressions of the six actors frame-by-frame. The calculated frame-by-frame motion energy was followed by averaging across frames. Next, we compared the obtained motion energy values with the ratings for each group via Spearman correlations. A similar bootstrapping analysis yielded estimates of confidence intervals for all dimensions across participants’ ratings.

**FIGURE 6:**
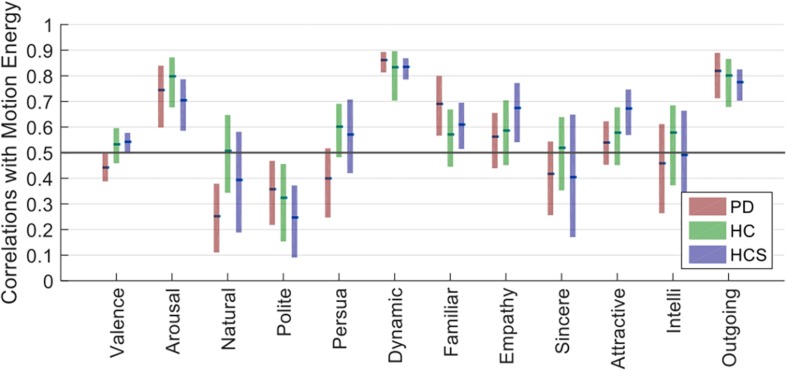
Optical flow estimate correlations with 12 dimensions for PD, HC, and HCS.

As shown in Figure 6, dynamic, arousal, and outgoingness have high correlation values for all three participant groups (*r* > 0.7), which is to be expected since these all relate to activity, i.e., movement actions in the facial expressions. Although there is some predictive power of motion energy for the other dimensions, their average values are much lower. Similar to the within-group reliability analyses, confidence intervals for all three groups overlap for the 12 dimensions, showing similar correlations with motion energy irrespective of age or prevalence of Parkinson’s.

Overall, this analysis shows that three rating dimensions carry information from low-level motion cues (i.e., the amount of movement of the face). Critically, the contribution of these motion cues to the ratings does not differ across the participant groups, showing that the decreased across-group correlations observed in Figure 4 are not directly attributable to differences in lower-level motion processing.

### Analysis of Individual Expressions

The final analysis was done on individual expressions based on the aforementioned positivity bias as a robust age effect in facial expression processing. We therefore evaluated absolute rating differences across the 12 jointly rated dimensions for the three participant groups using *t*-tests (Bonferroni-corrected for multiple comparisons). To investigate both disease and positivity effects, we separated the expressions into two subsets of negative expressions and positive expressions. This division was done based on the overall median of the valence ratings across all groups with expressions above the median being part of the positive set and expressions below the median being part of the negative set – overall 57 of the videos were determined as negative, whereas 27 were determined as positive.

Comparisons for the negative set are shown in Table 3 (significant results are shown in bold). As can be seen, ratings for valence differ between the young HCS and the older healthy control HC group. Naturalness and persuasiveness ratings differ between HCS and the older two groups (HC and PD). Empathy and sincerity ratings differ between HCS and HC groups (with a tendency for the PD group). Overall, we find fewer differences between the two older participant groups, compared to differences of either older group to the younger control group.

**TABLE 3:** Results of the statistical tests comparing ratings for negative expressions only across dimensions and different participant groups.

	**PD vs. HC**	**HCS vs. HC**	**PD vs. HCS**
Valence	n.s.: *p* = 0.427, *t*(38) = −0.802	**s.: *p* = 0.002, *t*(38) = 3.280**	n.s.: *p* = 0.024, *t*(38) = 2.358
Arousal	n.s.: *p* = 0.710, *t*(38) = 0.375	n.s.: *p* = 0.108, *t*(38) = −1.645	n.s.: *p* = 0.236, *t*(38) = −1.204
Natural	n.s.: *p* = 0.169, *t*(38) = −1.402	**s.: *p* = 0.000, *t*(38) = 5.140**	**s.: *p* = 0.002, *t*(38) = 3.290**
Polite	**s.: *p* = 0.001, *t*(38) =** −**3.574**	n.s.: *p* = 0.318, *t*(38) = 1.012	n.s.: *p* = 0.007, *t*(38) = −2.879
Persuasiveness	n.s.: *p* = 0.144, *t*(38) = −1.490	**s.: *p* = 0.001, *t*(38) =** −**3.756**	**s.: *p* = 0.000, *t*(38) =** −**4.444**
Dynamic	n.s.: *p* = 0.765, *t*(38) = −0.301	n.s.: *p* = 0.172, *t*(38) = −1.392	n.s.: *p* = 0.169, *t*(38) = −1.402
Familiar	n.s.: *p* = 0.723, *t*(38) = 0.357	n.s.: *p* = 0.026, *t*(38) = −2.318	n.s.: *p* = 0.106, *t*(38) = −1.654
Empathy	n.s.: *p* = 0.756, *t*(38) = 0.313	**s.: *p* = 0.001, *t*(38) =** −**3.581**	n.s.: *p* = 0.010, *t*(38) = −2.710
Sincere	n.s.: *p* = 0.031, *t*(38) = 2.243	**s.: *p* = 0.000, *t*(38) =** −**5.761**	n.s.: *p* = 0.010, *t*(38) = −2.703
Attractive	**s.: *p* = 0.001, *t*(38) =** −**3.581**	n.s.: *p* = 0.735, *t*(38) = 0.342	**s.: *p* = 0.002, *t*(38) =** −**3.417**
Intelligence	n.s.: *p* = 0.114, *t*(38) = −1.617	n.s.: *p* = 0.685, *t*(38) = 0.408	n.s.: *p* = 0.180, *t*(38) = −1.366
Outgoing	n.s.: *p* = 0.033, *t*(38) = −2.209	n.s.: *p* = 0.319, *t*(38) = −1.009	**s.: *p* = 0.001, *t*(38) =** −**3.434**

*s., significant after Bonferroni correction (boldface); n.s., non-significant.*

**FIGURE 7:**
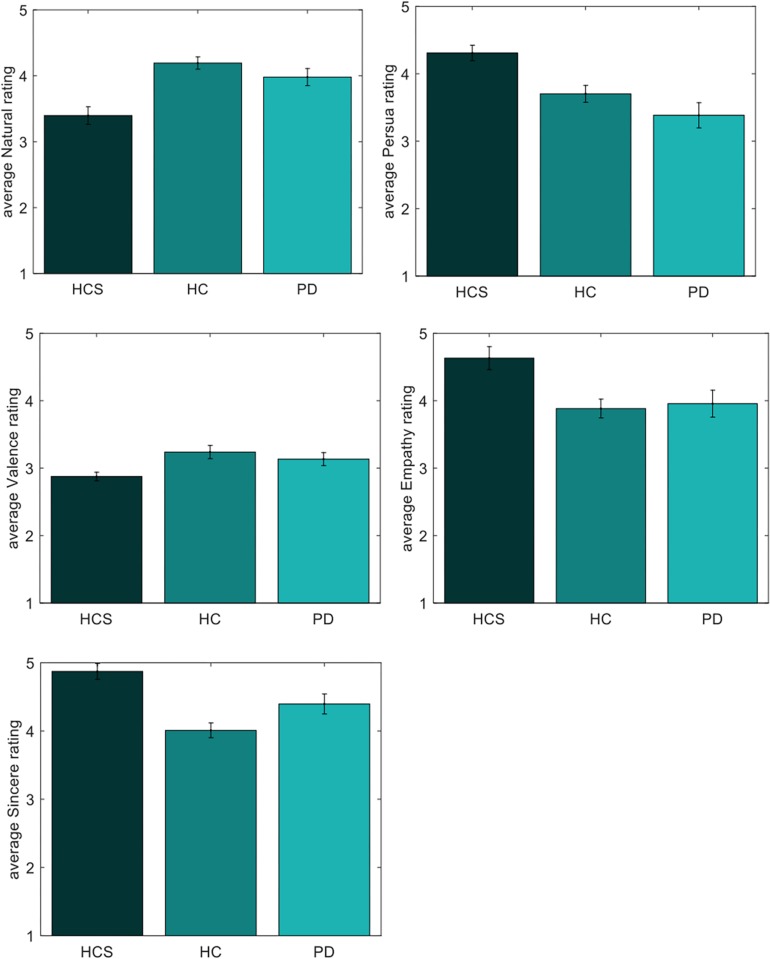
Individual ratings of negative expressions for naturalness, persuasiveness, valence, empathy, and sincerity.

As shown in Figure 7, the younger group assesses negative facial expressions as more natural, persuasive, sincere, and empathic compared to older participants. Similarly, older participants have a tendency to perceive the negative expressions as more positive compared to the younger participants – a finding that is compatible at first glance with the positivity effect.

In contrast, results for the positive expressions indicate no significant differences for any of the three tested participant groups PD-HC, HCS-HC, and PD-HCS.

## Discussion

How facial expression processing may be affected by the presence of PD or age-related factors has so far been addressed mainly in the context of recognition experiments on the so-called universal expressions. In this paper we aimed to investigate how a wider range of communicative facial expressions is evaluated in three different participant groups across factors of PD and age using a rating task: PD patients, an age-matched control group (HC) and another, younger participant group (HCS). This experimental setup allowed us to look at the effects of both PD (contrasting the PD and the HC group) and age as a measurement of age-related differences (contrasting the HC and the HCS group).

We found that all three groups were consistent in their rating patterns overall, showing that the evaluative dimensions were interpretable with robust rating performance. In order to compile the large set of evaluative dimensions into associations and to exploit potential correlational structure in that data, we next performed a factor analysis of the ratings. We found that inter-relation of factors overall was similar across three participant groups with common factor clusters that we were able to identify as related to authenticity, positivity, activity, and attraction.

To investigate this pattern in more detail, the results comparing the rating patterns *across* the groups showed that the two older participant groups were more similar to each other than either of these groups (HC and PD) to the younger HCG. Specifically, among the 12 evaluative dimensions we tested, the factors of naturalness, persuasiveness, empathy, and sincerity showed significant differences in rating pattern for older compared to the younger groups. Since each group showed consistent rating patterns *within* the groups, this means that for the task of evaluating facial expressions, age is a more critical factor than prevalence of PD. In contrast to the previous studies reporting potential *recognition* deficits in PD, the present study did not find significant differences for *evaluation* of facial expressions between PD patients and older adults. Note that in this context, our results overall are therefore not fully compatible with the strong version of the facial feedback hypothesis, as this would have predicted differences of processing in the PD group compared to the control group. To validate this statement fully, however, it would be necessary to also measure the facial expression *production* of the two groups [as done, e.g., in Marneweck et al. (2014) in the context of a recognition task].

Concerning age-related differences in facial expression processing, the positivity effect has been identified as an age-related alteration in which increased attention is placed on positive information with weakened processing of negative information (Baumeister et al., 2001; Rozin and Royzman, 2001; Mather and Carstensen, 2003). This processing change happens in a motivational, goal-oriented manner rather than due to a decline in cognition due to aging (Li and Lindenberger, 1999; Labouvie−Vief et al., 2010). The positivity effect can be explained within the socio-emotional selectivity theory that describes a motivated life-span theory of emotional and cognitive goal selection within temporal boundaries as the aging progresses (Carstensen et al., 1999; Carstensen, 2006). A neuroimaging study by Ebner et al. (2012) highlighted the possible generator of the positivity effect in older adults as the prefrontal cortex, specifically the anterior cingulate and medial frontal gyrus. This region was also found to be engaged more in older adults compared to younger adults during processing of negative stimuli versus positive stimuli (angry vs. happy face, Williams et al., 2006). Other brain imaging studies have also implicated increased amygdala responses to positive stimuli in older adults (Mather et al., 2004; Carstensen, 2006).

Given the extensive literature on the positivity effect as an age-related factor, we next looked for evidence supporting this effect in the individual absolute ratings. In accordance with the positivity effect, we found significant differences in the valence dimension for one group of older participants – an effect that was present for negative expressions only, however, Rutter et al. (2019) reported decreased emotion sensitivity to negative emotions in older subjects, although the task in the mentioned study was discrimination, a very large subject number with minimal but target oriented amount of emotions confirms our result of positive approach to specific to negative expressions in older subjects. In addition, we also found differences in the dimensions of naturalness, empathy, sincerity, and persuasiveness for which the negative expressions were rated as more neutral by one or both older groups in comparison to the younger participants. In addition, we found that the two older groups differed in two dimensions with the PD group rating the expressions as less polite and less outgoing compared to the HC group.

Whereas the first finding (differences in valence) may speak in favor of the positivity effect, when taken together with the other, additional rating differences we arrive at a more cautious interpretation: given the rating differences for the non-valence dimensions that relate to factors of authenticity and empathy, we can interpret these results as showing that the older participant groups did not connect as well with the expressions as the younger participant group did. One possible explanation for this result could be that the facial expression displays used in the present study show expressions performed by people in their mid- to late-twenties. As has been shown by Ebner et al. (2011), face processing is also influenced by an own-age bias, that is, optimized processing of stimuli derived from peers of similar age (see also Anastasi and Rhodes, 2006; Harrison and Hole, 2009; He et al., 2011). Another explanation to this finding could be perceptual processing of the individual. Macchi Cassia (2011) suggested starting from early childhood, interaction with different age groups shape the perceptual sensitivity (old age = 60 and above). Therefore, peer interaction may play an advantageous role in the later years of adulthood and age-related stereotypes (Malatesta and Izard, 1984) due to the tendency to look longer at own-age faces (mean old age = 73.52 ± 8.39) (Ebner et al., 2011). Hence, although the overall factors in the rating results are similar across our participant groups, the age differences in some of the rating dimensions for the older participant groups may be driven by this own-age bias.

A potential mechanism behind the positivity effect in the aging brain is cited to be a decreased dopaminergic functioning (Bäckman et al., 2000, 2006; Nieuwenhuis et al., 2002). Given this assumption and provided that the effect of dopamine is relatively fast-acting, PD patients in our experiment should have exhibited a much stronger positivity effect compared to healthy older adults given their much reduced dopamine levels when being off their medication. Within the limits of the sample size in this study, however, we found no clear differences regarding valence processing of facial expressions between the PD and older healthy controls. The studies by Kan et al. (2002), Sprengelmeyer et al. (2003), Dujardin et al. (2004), and Suzuki et al. (2006) did find recognition deficits in PD patients, which was in these cases restricted mostly to impaired recognition of the disgust expression. Interestingly, several previous studies have shown that disgust is often misclassified as anger (as is the expression of fear as surprise, e.g., Jack et al., 2009; Du and Martinez, 2011), such that recognition accuracy of individual expressions without a look at the confusion pattern itself may not be the most reliable indicator of population differences. Additionally, our stimuli were presented in their natural, dynamic form as opposed to the static pictures employed in the above-mentioned studies. As, for example, Cunningham and Wallraven (2009) have shown, static and dynamic presentation of facial expressions yield different result patterns even during standard recognition tasks. Hence, we believe that for our evaluation-based task, the results on natural stimuli do not indicate large, significant processing differences dependent on the presence of Parkinson’s.

Among the 12 rating dimensions, two had reliable differences (naturalness and persuasiveness) for both older groups compared to the younger group with two additional dimensions having tendencies in a similar direction (empathy and sincerity). These findings confirm our previous analysis of the rating patterns, which indicated differences in similar dimensions related to authenticity. We also did find two differences in rating values between the two older groups (politeness and attractiveness), opening up two potential dimensions for which the presence of Parkinson’s may play a role and that would need to be investigated further. In general, however, it needs to be stressed that the analysis of *absolute* rating values in the absence of clear anchoring points has limitations (see section “Reliability of Ratings Within Groups”), leading us to suggest that the overall focus in interpreting the results should rest more on the analysis of the ratting *patterns* as conducted in the other parts of this manuscript.

A final, confirmatory analyses concerned potential low-level correlates of the ratings themselves: using optic flow processing, we found that motion energy was able to account for significant variability in activity-related rating dimensions (dynamics, outgoingness, and arousal). Importantly, this analysis highlighted no clear differences between groups in any rating dimension, such that we assume that lower-level processes contributed in similar ways to ratings across both age and disease factors.

In the context of the present study, a recent review paper summarized the existing evidence on the effects of PD on facial expression processing in depth (Argaud et al., 2018). The authors review facial expression processing in PD using different tasks showing that 64% out of a total of 97 papers reported some sort of deficit. Most of the studies used *discriminative, forced-choice-type tasks* and except for one study (Kan et al., 2002, who found a larger deficit for fear and a smaller for disgust expressions), all studies used *static* stimuli of facial expressions – an issue highlighted also in the review paper. Out of the six studies using rating tasks, one reported no deficit (Adolphs et al., 1998), however, again only static expressions were used. Importantly, the rating tasks focused on ratings of expression of *intensity*, i.e., a typical question would be: “how much does the displayed expression portray the ‘disgust’ emotion: 0 = not at all, 5 = completely’. This is different from the task employed in the present study focusing on evaluating the content of the displayed facial expression according to a set of descriptive adjectives (inspired by Fontaine et al., 2007; Castillo et al., 2014), which we believe can add a different dimension to facial expression processing.

Given the above discussion about the existing state of evidence on deficits in facial expression processing in Parkinson’s, our study suggests that differences between a control population and a patient population may be much smaller when (a) using dynamic stimuli and/or (b) using an evaluative rating task. With respect to the first point, further studies are needed to address the issue of dynamicism in different types of tasks with larger sets of stimuli (e.g., Cunningham and Wallraven, 2009). With respect to the second point and given that relatively few rating studies have been conducted so far, it will be also interesting to extend our study to test the same (non-demented) control and patient populations with both a forced-choice recognition task as well as a rating task in order to investigate effects of task type and task demands in the context of Parkinson’s prevalence.

In summary, the findings of our study show that evaluation of natural, dynamic facial expressions results in consistent, and broadly similar patterns across both younger and older participants that capture aspects of authenticity, positivity, dynamics, and attraction. Differences between the groups were discernible for several rating dimensions related to the authenticity factor, with both older groups perceiving especially negative expressions as more positive, but also as less natural, persuasive, and empathic. We interpreted these results as coming from a possible own-age bias that will need to be investigated further in future studies.

Importantly, although Parkinson’s is known to have severe effects on a wide variety of perceptual and cognitive tasks, our experiments have not indicated that it has such effects during the evaluation of dynamic emotional and conversational expressions. Interestingly, the argument has been made that such a task may be much more ecologically valid to real-life situations than forced-choice recognition of static snap-shots of facial expressions (e.g., Russell, 1994; Argaud et al., 2018). As such the results may be due to resilient cognitive processes involved in affective evaluation of moving faces even in the process of such a major change in brain processing. We hope that our study can pave the way for investigations of both PD and aging effects in general using a richer set of tools that will lead to a deeper understanding of its effects on the perceptual and cognitive processes in the brain.

## Data Availability Statement

The datasets generated for this study are available on request to the corresponding author.

## Ethics Statement

All individuals provided written informed consent to participate in this study and the nature of the study was explained to each participant. All participants were paid for their participation and were informed about the possibility to stop the experiment anytime they would like to. The experiment was conducted in accordance with the Declaration of Helsinki 1963 and approved by the Ethics Committee of Korea University (AS17006).

## Author Contributions

DD and CW conceived and designed the experiments and analyzed the data. DD conducted the experiments. JK and D-YK were responsible for recruiting and supervising Parkinson’s patients. All authors wrote the manuscript.

## Conflict of Interest

The authors declare that the research was conducted in the absence of any commercial or financial relationships that could be construed as a potential conflict of interest.
